# Perspectives and Challenges of COVID-19 with Obesity-Related Cancers

**DOI:** 10.3390/cancers15061771

**Published:** 2023-03-15

**Authors:** Maria Dalamaga, Narjes Nasiri-Ansari, Nikolaos Spyrou

**Affiliations:** 1 Department of Biological Chemistry, School of Medicine, National and Kapodistrian University of Athens, 75 Mikras Asias, 11527 Athens, Greece; 2 Tisch Cancer Institute Icahn School of Medicine at Mount Sinai, 1190 One Gustave L. Levy Place, New York, NY 10029, USA

The emergence of COVID-19 has created an unprecedented threat worldwide, involving overwhelmed health-care systems in the majority of countries [1,2]. Cancer screening and delivering care in oncologic patients has been a challenging task based on the balance between the risk of mortality from untreated malignancy versus the risk of severity of COVID-19 and death in immunocompromised individuals [3].

The COVID-19 pandemic has taken place at a time when the worldwide prevalence rate of overweight and obesity is greater than 39% and 13%, respectively, for adults, and cancer is a leading cause of death globally based on recent WHO data [4,5]. Obesity has become a major global public health concern related to a variety of disorders, including metabolic syndrome, type 2 diabetes mellitus, cardiovascular disease, nonalcoholic fatty liver disease, polycystic ovary syndrome, autoimmune disorders and cancer [6,7,8,9,10,11,12,13].

Interestingly, a growing body of evidence has shown that obesity and increased visceral fat are strongly and independently linked with adverse outcomes and death due to COVID-19 [14,15,16,17,18]. Moreover, there is convincing evidence that excess body weight is associated with an increased risk for cancer of at least 13 anatomic sites, including endometrial, esophageal, renal and pancreatic adenocarcinomas; hepatocellular carcinoma; gastric cardia cancer; meningioma; multiple myeloma; colorectal, postmenopausal breast, ovarian, gallbladder and thyroid cancers [19,20,21].

Two umbrella reviews and meta-analyses on the association between pre-existing health conditions and severe outcomes from COVID-19 have shown that other than diabetes, obesity, heart failure, chronic obstructive pulmonary disease and dementia, active cancer was also linked with a higher risk of death, particularly in Europe and North America [22,23]. Oncologic patients may be more susceptible to SARS-CoV-2 infection. Evidence is mixed regarding COVID-19 severity in cancer patients [24,25,26], while COVID-19 outcomes in these patients are improved with an earlier diagnosis, the availability of effective COVID-19 vaccines, the advent of the omicron outbreak and the timely use of anti-viral therapy [24,27]. Despite all the abovementioned factors, most studies have highlighted an elevated risk of severe COVID-19 in patients with active cancer, particularly in non-vaccinated individuals [22,23,25,28,29]. The risk may vary by type and stage of cancer and treatment. Particularly, older age, hematologic malignancies, lung cancer, advanced or progressive cancer, active chemotherapy, comorbid conditions, including obesity, and smoking are associated with severe COVID-19 [30,31,32,33,34,35,36,37].

COVID-19 had devastating effects on cancer patients, with an immense number of missed diagnoses and delayed treatments attributed mainly to patient’s reluctance to seek medical care and to health systems under pressure [38]. Throughout the pandemic and particularly during the first and second phases, there were significant delays in cancer screening and surveillance that resulted in delayed diagnoses, particularly in obesity-associated cancers, an increased rate of patients diagnosed in an emergency setting, a plethora of diagnoses of advanced stage malignancies with higher tumor burden as well as delays in the treatment of patients with newly diagnosed cancers [39,40,41,42,43,44]. In the initial phase of the pandemic, collective data from 200 sources in 17 European countries have shown that an estimated 100 million screening tests were not performed while up to 50% of all cancer patients were affected by treatment delays [45].

Generally, therapeutic management of oncologic patients with COVID-19 is similar to that used for the general population. In the outpatient setting, available treatment options comprise antiviral agents, such as nirmatrelvir-ritonavir, remdesivir and molnupiravir, as well as monoclonal antibodies active against prevalent variants [46,47]. Persistent SARS-CoV-2 infection may uncommonly occur in immunocompromised patients, particularly those with severe B-cell depletion due to cancer treatment (e.g., rituximab, hematopoietic cell transplantation, etc.) [46,48,49].

Overall, when cancer treatment is indicated according to consensus guidelines, it should be administered without delay [50]. In most hematologic cancers, the efficacy of treatment favors the adoption of curative approaches, despite the infectious risk of COVID-19; however, decisions should be made on a case-by-case basis [51]. In patients who have tested positive for SARS-CoV-2, immune checkpoint inhibitors (ICIs) should be postponed until recovery [50]. Transplantation/cellular therapy should be delayed for 2–3 weeks and until patients are asymptomatic; however, decisions should be made on a case-by-case basis (e.g., prolonged viral shedding should be taken into consideration) [52]. In cases where treatment is not urgent as in indolent lymphomas, treatment can be deferred until nasopharyngeal sampling is negative and symptoms have resolved; less immunosuppressive therapies can also be prioritized [53]. Moreover, in the absence of urgent treatment indication, an individual treatment deferral after anti-SARS-CoV-2 vaccination (at least one injection) may be considered [53]. Therapeutic decisions to proceed with systemic therapies should be preceded by a multidisciplinary discussion, risk/benefit analysis and discussion with the patient.

Up-to-date COVID-19 vaccination is recommended to all patients with active or prior malignancy in order to prevent severe SARS-CoV-2 infection. Data have indicated that vaccine efficacy is relatively diminished in patients with active cancer in comparison to individuals without cancer, especially in patients with hematologic malignancies, and particularly those on anti-CD20 antibody treatment [54,55,56]. Patients with hematologic malignancies present lower seroconversion rates post-vaccination, potentially leading to inferior COVID-19 outcomes despite vaccination, and an elevated risk for breakthrough infections [57,58,59]. Figure 1 depicts the main challenges of COVID-19 associated with cancer.

This Special Issue places emphasis on the challenges surrounding COVID-19 and obesity-related cancers, including, but not limited to: risk factors of severe COVID-19 in cancer patients; complications of COVID-19 in cancer patients; challenges in cancer treatment and surgery; guidelines for cancer care during COVID-19; delayed diagnosis and suboptimal cancer management; impact of COVID-19 on cancer screening, early diagnosis and cancer presentation; immune response after SARS-CoV-2 infection and vaccination in cancer patients; psychological distress among cancer survivors; telehealth in cancer care; role of diet and nutraceuticals in the prevention of severe COVID-19 and cancer as well as effects of COVID-19 pandemic on cancer research.

Although SARS-CoV-2 mainly affects the lower respiratory tract, it can initiate system-wide pathophysiological alterations and dysfunctional immune responses. Habibzadeh et al. reviewed the potential effects of autophagy on cancer development and outcomes [60]. Various SARS-CoV-2 proteins interact with different components of the cellular autophagy pathway and could lead to long-term defects in the cellular autophagy machinery. The authors hypothesize that these defects could mediate alterations in cell proliferation, immunity against cancer cells, cell metabolism and cancer drug efficacy that could eventually impact on carcinogenesis and responsiveness to cancer therapy.

The lack of medications for COVID-19 in the early phases of the pandemic led the public to seek alternative therapies, including nutraceuticals. Bader-Larsen et al. evaluated the safety of “anti-COVID-19” nutraceuticals in patients with cancer [61]. They reported that the use of vitamin C, vitamin D and selenium supplements is likely safe at typically recommended doses. However, they cast caution on the use of omega-3 fatty acids and zinc, as potential risks may outweigh the benefits derived from their use.

Almasri and colleagues reviewed the evidence on the immune response rates and safety profiles of COVID-19 vaccines in patients with hematologic and solid malignancies [62]. In comparison to the general population, lower seropositivity following vaccination was associated with malignancy as well as with hematologic malignancy compared to solid cancers. Patients receiving active chemotherapy, radiotherapy and immunosuppressive therapy generally displayed lower seropositivity rates compared to healthy controls, a phenomenon not observed in patients who received checkpoint inhibitors, endocrine therapies and cyclin-dependent kinase inhibitors. Nevertheless, cancer patients demonstrated the ability to mount safe and effective immune responses to COVID-19 vaccines. Adverse events were comparable to those of the general population; however, inflammatory lymphadenopathy following vaccination was commonly reported and may be mistaken for a lymphadenopathy of malignant etiology. Finally, the authors suggested that vaccination should be promoted, and reluctance to receive it should be addressed in this population.

The SARS-CoV-2 pandemic brought multi-dimensional disruptions in oncology. Ali and Riches reviewed the changes that affected the care of cancer patients as well as clinical trials [63]. The pandemic disrupted routine cancer screenings and decreased the potential for early cancer detection, resulting in many thousands of missed cancer diagnoses. Initially, clinical trials were significantly impacted, resulting in the cessation of trial initiation and recruitment, particularly in oncology clinical trials. Clinical trial practices and protocols underwent significant changes, including a reduction in in-person appointments, use of telemedicine and remote monitoring systems, to minimize the risk of exposure to the virus. Cottenet et al. studied the effect of obesity on the risk of intensive care unit (ICU) admission, severe complications and in-hospital mortality, in 992,899 cancer patients hospitalized with or without COVID-19 from the French national administrative database [64]. The authors found that obese cancer patients had an increased risk of admission to the ICU and severe complications, regardless of the type of obesity. Severe obesity was associated with an increased risk of in-hospital mortality, particularly in patients with hematological cancers. This study showed that among hospitalized cancer patients with or without COVID-19, increased vigilance is needed for obese patients, both in epidemic and non-epidemic periods. Lastly, Sereno et al. conducted a retrospective study in 943 cancer patients, of whom 83 (11.3%) were neutropenic and infected with COVID-19, looking at the effect of G-CSF treatment on outcomes during the first phase of the pandemic [65]. They observed a significant effect of the number of days of G-CSF treatment on severe disease and mortality, suggesting that prolonged treatment could be disadvantageous for these patients.

The management of many diseases was altered after the COVID-19 outbreak. Obesity has a great impact on COVID-19 patients’ outcome while it affects the etiopathogenesis of various types of cancers, including melanoma, thyroid (TC) and hematological cancers. Cariti et al. critically evaluated whether screening, diagnosis and treatment of melanoma have been changed by the COVID-19 pandemic as well as the implications of these changes [66]. The authors found that during the pandemic, there was a delay in the diagnosis of new melanoma cases. After detection, the management of early-stage and advanced melanoma cases was fully guaranteed, whereas the follow-up visits of disease-free patients were delayed or replaced by teleconsultation when possible. The authors reported their valuable experience in the management of patients with melanoma during the COVID-19 pandemic, in a dermatological clinic in Northern Italy.

The interplay between obesity, TC and COVID-19 was extensively evaluated by Deligiorgi et al. [67]. Obesity is associated with an increased risk of differentiated TC (DTC). The authors summarized exciting, published data on the association between COVID-19 and TC into four sections: (i) the interrelationship between obesity, immunity, inflammation and oxidative stress, underlying this association; (ii) the challenging management of (D)TC in the COVID-19 era; (iii) the impact of COVID-19 on (D)TC and vice versa; and (iv) the oncogenic potential of SARS-CoV-2. The researchers highlighted the importance of evidence-based, risk-stratified and consensus-based decision making for providing the safest premium-quality care for (D)TC patients, with or without COVID-19.

Both obesity and hematological cancers are well-known risk factors for severe SARS-CoV-2 infection. Tsilingiris and colleagues comprehensively summarized the evidence linking obesity to the development of hematological cancers as well as their interconnection with severe COVID-19. They analyzed various challenges associated with the management of patients suffering from hematological cancers, including prevention, diagnosis and therapeutic approaches [46]. They thoroughly highlighted the unsolved issues and challenges that need to be overcome in the ongoing COVID-19 pandemic. The co-existence of obesity and hematological malignancies in the frame of SARS-CoV-2 infection may theoretically confer an adverse prognosis. Recent global-scale data have shown that the diagnosis of multiple myeloma has been missed or delayed in the early COVID-19 era compared with the previous year, while a decreased survival among newly diagnosed cases has been noted. The field of hemato-oncology has already overcome the initial blow to a great degree and adjusted to the new reality brought forward by COVID-19. Nonetheless, there is a need for continued appraisal and update of numerous aspects of therapeutic management. Many issues need to be determined, including the optimal timing of hematology–oncology therapy initiation, the post-treatment follow-up and the timely therapeutical planning, including allogeneic hematopoietic stem cell transplantation in the case of infections that emerge during the active medical therapy.

Apart from the abovementioned therapeutic approaches in oncologic patients during the COVID-19 pandemic, special attention must be given to patients who need oncological surgery because of the modification of treatment guidelines, the reduced access of patients to healthcare resources and the postponement of surgery. Prodromidou and colleagues studied how Enhanced Recovery after Surgery (ERAS) protocols may provide optimal management of patients with obesity and malignancy during the COVID-19 pandemic, with a special focus on patients who required surgery for gynecologic malignancy [68]. They highlighted the importance of the establishment of protective measures before surgery in order to reduce the exposure of cancer patients to SARS-CoV-2 while simultaneously improving vaccination access. Finally, the authors emphasized that ERAS protocols may play a crucial role in the management of patients with gynecologic cancer based on their efficiency in many surgical fields, such as general surgery and orthopedics, during the COVID-19 pandemic.

Breast cancer patients and caregivers were also tremendously affected by the healthcare crisis in multiple domains and ways. Petropoulou et al. summarized the challenges in breast cancer management and the subsequent implications in clinical practice [69]. Similar to other obesity-related cancers, screening, diagnosis and surgical intervention were the most affected domains, causing a serious psychological burden to patients. The aftermath of diagnostic and surgical delays is yet to be assessed, while alterations in the treatment plans and the introduction of new therapeutic schemes, such as the extensive use of neoadjuvant hormonal therapy and the significant reduction in the first-line surgical approach, may open a novel era in the management of breast cancer.

Furthermore, the COVID-19 pandemic resulted in changes in surgical practice involving colorectal malignancies. In a systemic review and meta-analysis of ten studies including 26,808 patients, Pararas et al. evaluated the impact of the COVID-19 pandemic on the management of colorectal cancer, the third-most-common cancer worldwide, which is also associated with obesity [70]. The authors found that the number of patients presenting with metastases during the pandemic was significantly increased, with no differences regarding the extent of the primary tumor and the nodal status. In addition, patients with colorectal cancer during the pandemic were more likely to undergo palliative interventions or receive neoadjuvant treatment.

Finally, we expect that these manuscripts contributed by experts in this field will provide useful resources to physicians and scientists interested in the interplay of COVID-19 and obesity-related cancer, as well as the development of relevant preventive and therapeutic strategies.

## Figures and Tables

**Figure 1:**
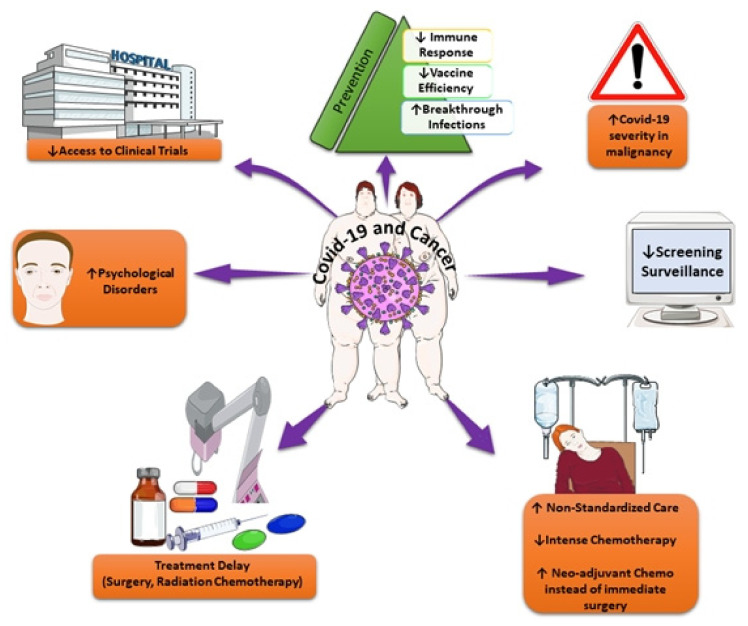
Main challenges of COVID-19 associated with cancer. This image was derived from the free medical site http://smart.servier.com/ (accessed on 2 March 2023) by Servier, licensed under a Creative Commons Attribution 3.0 Unported License.
